# Imaging the implant-soft tissue interactions in total knee arthroplasty

**DOI:** 10.1186/s40634-016-0061-5

**Published:** 2016-10-03

**Authors:** Michel P. Bonnin, Tom Van Hoof, Arnoud De Kok, Matthias Verstraete, Catherine Van der Straeten, Mo Saffarini, Jan Victor

**Affiliations:** 1 Centre Orthopédique Santy, 24 Av Paul Santy, Lyon, France; 2 Hopital Privé Jean Mermoz, 55 Av Jean Mermoz, 69008 Lyon, France; 3 UZ Gent, De Pintelaan, 185 Gent, Belgium; 4 Accelerate Innovation Management, Rue de Hollande 4-6, 1204 Geneva, Switzerland

**Keywords:** Painful TKA, Soft tissue impingement, Popliteus tendon, 3D printing

## Abstract

**Background:**

In Total Knee Arthroplasty (TKA), residual pain may be secondary to soft tissue impingements, which are difficult to visualize around chromium-cobalt implants using medical imaging, so their interactions remain poorly understood. The goal of this work was to establish a protocol for in-vitro imaging of the soft tissues around TKA, usable during throughout the range of motion (ROM).

**Methods:**

The full size range of a commercially available TKA prosthesis was manufactured by 3D-printing in non-magnetic and non-radiopaque polymer and implanted in 12 cadaveric knees. The relations between these implants and the soft tissues (Popliteus tendon, Medial and Lateral Collateral Ligament, Patellar and Quadriceps tendons) were analyzed, using MRI (5 embalmed specimens) and CT scans after injection of the tissues with barium-sulfate (3 embalmed and 4 fresh-frozen specimens).

**Results:**

Both MRI and CT scans enabled good identification of the soft tissues before TKA implantation. MRI produced minimal loss in signal and contrast, and neither the low temperature nor the embalming fluids compromised image quality. CT scans were more precise after TKA implantation, particularly the borders of the implant and the differentiation of soft tissues. Full ROM investigation, manual segmentation and three-dimensional reconstructions were possible only with the CT scan.

**Conclusion:**

The experimental approach described in this study was successful in visualizing the interactions between the soft tissue and the implants before and after TKA and during the full ROM. The coordinate system allows to localize precisely the different anatomic structures and to quantify any change due to prosthetic implantation.

## Background

In Total Knee Arthroplasty (TKA), residual pain and poor functional outcomes can be due to impingements between prosthetic components and soft tissues such as the Popliteus tendon (popliteus) (Barnes & Scott 1995; Kazakin et al. 2014), the Patellar tendon (PT) (Argenson et al. 2005), the iliotibial band (ITB) (Luyckx et al. 2010) or the Medial Collateral Ligament (MCL) (Bonnin et al. 2015; Bonnin et al. 2013). Impingements can be secondary to prosthetic overhang or component malposition (Allardyce et al. 1997; Barnes & Scott 1995; Bonnin et al. 2015; Bonnin et al. 2013; Kazakin et al. 2014; Mahoney & Kinsey 2010) but may also occur in well-sized and well-positioned prostheses (Allardyce et al. 1997). Optimizing bone-implant fit is therefore a concern for surgeons, engineers and manufacturers, and precise knowledge of the interactions between soft tissues, bone contours and prosthetic components during full range of movement would be useful.

In-vivo imaging of the soft tissues around metallic implants is challenging. Magnetic Resonance Imaging (MRI) allows high quality explorations if implants are made from non-magnetic alloys like titanium but it provides poor quality images with Chromium-Cobalt alloys, commonly used in TKA, (Sutter et al. 2013). Even when using metal artifact reduction sequences (M.A.R.S.), soft tissue visualization around TKA remains of poor quality (Hayter et al. 2011; Naraghi & White 2006; Olsen et al. 2000). Recent investigations performed in specialized centers still report a lack of accuracy for visualization the soft tissue (Li et al. 2016a) and the polyethylene (Li et al. 2016b) after knee prostheses. Computed Tomography (CT) is widely used in patients with TKA, but does not enable accurate identification of soft tissues, particularly in the presence of metallic components due to scattering (Ho et al. 2012). Ultrasonography can be used for clinical purposes and allows dynamic explorations but it is hardly used for precise anatomic investigations (Math et al. 2006).

Cadaver dissections help to understand the relations between bone and soft tissues, but correct visualization requires aggressive dissections, which compromise the native anatomy and enable only qualitative assessments. The use of CT scans or MRI with cadaver specimens could avoid such large dissections and enable quantitative measurements of soft tissue displacements and impingements, but the above-mentioned difficulties with in-vivo MRI or CT scan persist.

The purpose of this work was to optimize a technique for in-vitro imaging of the soft tissues around TKA usable during the full range of motion. In order to circumvent the difficulties encountered with chromium-cobalt implants, we obtained from a manufacturer the full range of a commercially available prosthesis made from non-magnetic and non-radiopaque polymer. We analyzed the relations between these plastic implants and the surrounding soft tissues, using MRI and CT scan after cadaveric implantation. We compared the imaging technique depending on the radiological technique (MRI or CT scan) and the type of specimen (embalmed or fresh-frozen). We therefore asked several questions: Is it possible to obtain a good vision of the soft tissues around such plastic implants using standard imaging techniques? Which technique between MRI or CT scan provides the best quality images? Which preparation of specimens between embalmed or fresh-frozen provides the best images?

## Methods

Twelve human cadavers donated for research by testament were used in this investigation and our institutional review board granted ethical approval for this study in advance (Reference number EC-2014/0847). None of the cadaver knees had history of previous surgery.

The investigation focused on the Popliteus Tendon (popliteus), the Lateral Collateral Ligament (LCL), the Medial Collateral Ligament (MCL), the Quadriceps Tendon (QT) and the Patellar tendon (PT). The ITB was excluded from this study due to technical difficulties outlined below. The visualization of these tissues was analyzed from full extension to maximum flexion. The implanted prosthesis was a copy of the HLS-KneeTech® (Tornier SA, Montbonnot, France) provided by the manufacturer and obtained using additive manufacturing technology with Fused Deposition Modeling FDM® using a Stratasys Dimension Elite™ (Eden Prairie, MN USA). The implants were made with a radio-opaque and non-magnetic polymer (Acrylonitrile butadiene styrene).

### TKA implantation

The prosthesis was postero-stabilized, with eight sizes available for the tibial component and ten for the femoral component. Implantation was done through a medial parapatellar approach and the patella was not resurfaced. We used the standard conventional instrumentation obtained from the manufacturer. The tibial cut was orthogonal to tibial axis and was done first. On the femur, the posterior cut was aligned parallel to the transepicondylar axis, the distal cut was orthogonal to the femoral mechanical axis and a gap-balancing technique was used. Cementation was done with polyester free from barium sulfate (Polyester Demaere, Brussel, Belgium).

### Imaging techniques

Three different preparations and imaging techniques were used. In group I (5 knees), MRI was performed on cadavers embalmed with the Thiel technique (Fessel et al. 2011; Thiel 1992). In group II (3 knees), CT scan analysis was also done on Thiel embalmed cadavers. In group III (4 knees), the CT scan was done on fresh frozen cadavers. The 12 cadaver specimens were initially intended to form 3 equal groups, but the challenges faced with MRI settings required the use of an additional specimen, which resulted in unequal group sizes. The embalmment technique described by Thiel in 1992 (Thiel 1992) intended to preserve tissue flexibility and joint mobility compared to classic embalmment techniques using glutaraldehyde or formaldehyde (Fessel et al. 2011), though the influence of the conductivity of the embalming fluids on the MRI signal and contrast remains controversial (Gueorguieva et al. 2014; Schramek et al. 2013).

### Group I - MRI imaging

A 3-Tesla MRI (Siemens Sensation, Munich, Germany) was used to scan the knees before and after TKA implantation. Due to the small diameter of the MRI tube, transections at the mid-shaft of the femur and the tibia were necessary to achieve full flexion of the knee. The knees were scanned in lateral decubitus position before and after TKA implantation, from full extension to full flexion in 20° increments between each position, measured with a goniometer. Particular attention was paid to maintain the room temperature at 20 °C during the entire process (Kobayashi et al. 2010).

### Groups II and III - CT scan imaging

With this technique a contrast medium was injected in the soft tissues in a first step. The popliteus and the LCL were approached via a lateral incision. After incision of the iliotibial band, the popliteus and the LCL were identified. The QT and the PT were approached using a medial subvastus incision. The MCL was approached from the anterior skin incision after subcutaneous dissection. The superficial fibers of the MCL were exposed from their epicondylar insertion to their distal tibial insertion. After exposure, the contrast medium - a mixture of glycerol (70 %) and barium sulfate (30 %) - was injected in these tissues using a previously described technique (Van Hoof et al. 2013): A thin needle (0.45 mm × 23 mm) was inserted between the collagen fibers of the explored tissue, and the solution was injected with mild pressure until leakage occurred at the injection site. Pieces of gauze swabs were wrapped around adjacent structures in order to prevent contamination of possible leaking contrast solution. The injection needle was then directed towards the insertion sites until the contact point with the bony surface. On this spot, a small bolus of contrast was injected and this was repeated covering the complete insertion area (Van Hoof et al. 2013). It is worth noting that the ITB was inaccessible to this technique due to its thin structure.

The barium concentration had been determined during a prior investigation where different concentrations from 30 to 90 % were tested, and 30 % appeared to be optimal for good visualization with minimal scattering (Van Hoof et al. 2013). After injection, the skin and subcutaneous tissues were meticulously closed.

The knee was then scanned using a 64-slice multidetector CT scanner (Siemens Sensation, Munich, Germany). Scans were performed with the full body tilted in lateral decubitus using 0.6 mm thick slices from extension to full flexion, by 20° increments controlled with a goniometer. The knee was scanned again with the same technique after TKA implantation.

### Analysis of the DICOM images

DICOM images were analyzed using OsiriX® software (Pixmeo SARL, Bernex, Switzerland) with 3D multiplanar reconstructions. The quality of the images obtained in the three groups was compared using 10 criteria, taking account of the bone visualization, the capacity to investigate the full range of motion, the visualization of the implants and the visualization of the soft tissues in contact with the implants. The four medical doctors evaluated all images blindly (two senior orthopaedic surgeons, one resident orthopaedic surgeon, and one rheumatologist who independently ranked each criterion from 0 to 5.

**Fig. 1:**
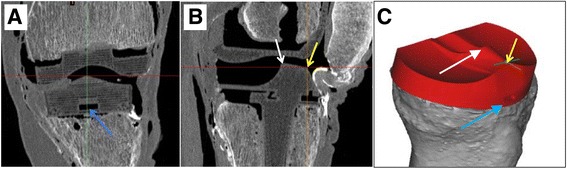
The STL files are imported in Mimics® and are fused with the raw images of the implants. Specific landmarks of the implants are used to do the manual fusion. On the tibia, three landmarks were used: (**a**) the anterior removal indentation of the polyethylene (*blue arrow*), (**b**) the most anterosuperior point of the polyethylene (*yellow arrow*), and the posterosuperior point of the cam (*white arrow*). The corresponding landmarks of the STL implant were then matched manually (**c**)

**Fig. 2:**
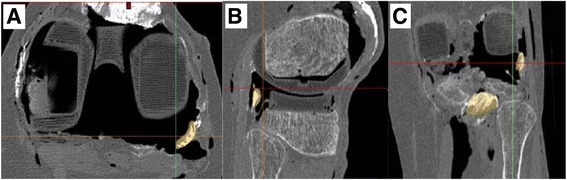
Segmentation of the soft tissues was done manually from the 3D multiplanar reconstructions. The popliteus tendon area is colored in yellow on the transverse (**a**), sagittal (**b**) and coronal (**c**) planes

Raw DICOM images were used to build three-dimensional reconstructions of bone, implants and soft tissues, using Mimics® software (Materialize®, Leuven, Belgium). First, Mimics® automatically built the bone masks. Second, to improve the accuracy of implant visualization, the Stereolithography (STL) files (3D Systems, Rock Hill, South-Carolina USA) provided by the manufacturer were matched with the postoperative reconstructions (Fig. 1). Third, the soft tissues were digitized by manual segmentation at each slice level (Fig. 2). Fourth, coordinates of digitized points were exported to spreadsheets and processed using Matlab® (MathWorks®, Natick, MA, USA), in order to analyze the position of all studied tissues during knee flexion and to quantify the potential displacements due to prosthetic implantation.

### Statistical analysis

Statistical correlations and tests were not performed for this cadaveric imaging study as its results were chiefly qualitative and involved no population-based data.

## Results

### Bone visualization

Comparative analysis of the imaging obtained in the three groups is summarized in Table 1.

**Table 1:** Comparison of the quality of the images obtained with the three protocols

	Evaluation (0 = worst, 5 = best)					
	MRI embalmed		CT embalmed		CT fresh-frozen	
	*Mean*	*Range*	*Mean*	*Range*	*Mean*	*Range*
Bone visualization						
Distinction of bone from soft tissues	2	*1 to 3*	3	*2 to 5*	4	*3 to 5*
Distinction cortical bone from cancellous bone	3	*2 to 4*	4	*2 to 5*	5	*5*
Similarity to conventional medical images	3	*2 to 4*	4	*2 to 5*	5	*5*
Prosthetic imaging						
Distinction of bone from implant material	1	*0 to 2*	4	*3 to 5*	4	*4*
Distinction of soft tissues from implant material	1	*0 to 2*	2	*1 to 3*	4	*4*
Soft tissue visualization						
Visibility of soft tissues along their entire length	3	*1 to 4*	3	*2 to 5*	3	*2 to 4*
Scattering/distortion	2	*1 to 3*	3	*2 to 5*	4	*4*
Range of motion						
Possibility to image the knee at all flexion angles	2	*0 to 3*	5	*5*	5	*5*
Mirroring/superposition	0	*0*	5	*5*	5	*5*
3D reconstructions						
Visibility of the entire knee	0	*0*	5	*5*	5	*5*
Total score	12		31		35

**Table 2:** Advantages and disadvantages between MRI and CT-scan

	CT	MRI
Advantages	• Fast • Not expensive • Possible for full lower limb • Possible on fresh-frozen cadavers • Adequate visualisation of bone and soft tissues	• Visualisation of all the soft tissues
Disadvantages	• Needs injection of barium-sulfate • Impossible to inject all structures • Contrast medium does not reach the bony insertions	• Time consuming • Expensive • Needs transection of femur and tibia to flex the knee • Poor image quality for bone and implant • Require temperature monitoring

**Fig. 3:**
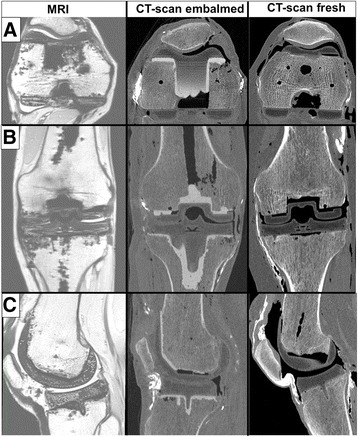
Comparative aspect of the operated knee with MRI on embalmed specimen-group I (*left*), with CT scan on embalmed specimen-group II (*middle*) and with CT scan with fresh specimen-group III (*right*). This picture shows the raw images obtained with OsiriX®, in the transverse plane (**a**), the coronal plane (**b**) and the sagittal plane (**c**)

With MRI only a minimal loss in signal and contrast was observed and neither the low temperature nor the Thiel embalming fluids seem to compromise the quality of the images. With CT scans, high quality images were obtained with a bony aspect close to what is observed in clinical practice. Interestingly, the quality of the images was similar when using Thiel embalmed and fresh cadavers (Fig. 3).

### Prosthetic imaging

Both MRI and CT scan provided good quality images of the plastic components, without scattering. Prosthetic imaging was more precise with the CT scanner analysis, particularly the borders of the implant were easier to individualize. Fusion of the DICOM images with the STL files was only possible using the CT scans because of better visibility of prosthetic contours.

### Soft tissue visualization

**Fig. 4:**
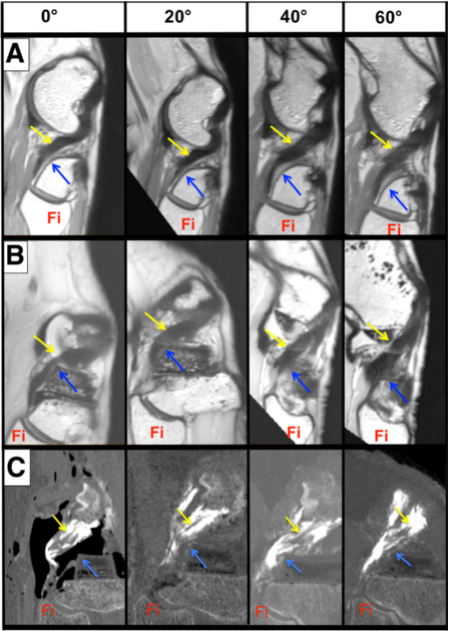
Visualization of the popliteus tendon (*yellow arrow*), the posterolateral corner of the lateral tibial plateau (*blue arrow*) and the head of the fibula (Fi) in an oblique parasagittal plane, during knee flexion. **a** (MRI) In the preoperative knee, the popliteus has very intimate relations with the posterolateral corner of the lateral tibial plateau during the all range of motion. **b** (MRI) Postoperatively, an impingement is visualized between popliteus and prosthetic plateau but the quality is poor (**c**) (CT-scan fresh-frozen specimen). The popliteus tendon (*yellow arrow*) is visible as well as the tibial prosthetic plateau (*blue arrow*)

**Fig. 5:**
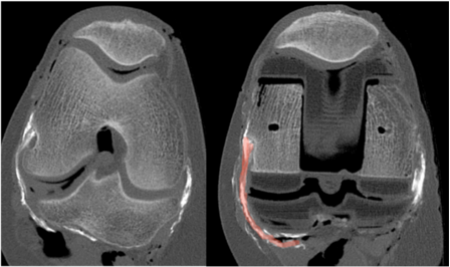
CT scan imaging of the popliteus tendon and MCL preoperatively (*left*) in a modified transverse plane. Postoperatively (*right*) the popliteus (*red*) and the MCL (*white*) are visible as well as their relationships with the bone and implant

**Fig. 6:**
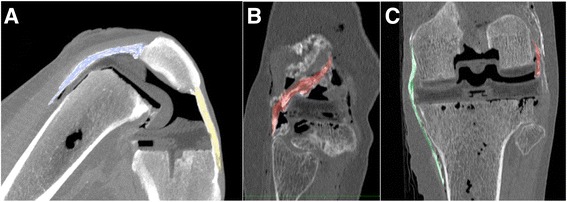
With CT scan on fresh specimen, the relationships between the implants and the soft tissues are visualized in the sagittal plane (quadriceps tendon in blue and patellar tendon in yellow) (**a**), in the oblique parasagittal plane (popliteus tendon in red) (**b**) and in the coronal plane (MCL in green and popliteus in red) (**c**)

In the native knee, visualization of the soft tissues was better with the high definition MRI. However after prosthetic implantation, the differentiation between implant and soft tissue was always weak and segmentation appeared to be unreliable using MRI (Fig. 4). Differentiation of soft tissues from the implants was better using CT scans on fresh frozen specimens, both at the femur (Fig. 5) and the tibia (Fig. 6).

### Range of motion

**Fig. 7:**
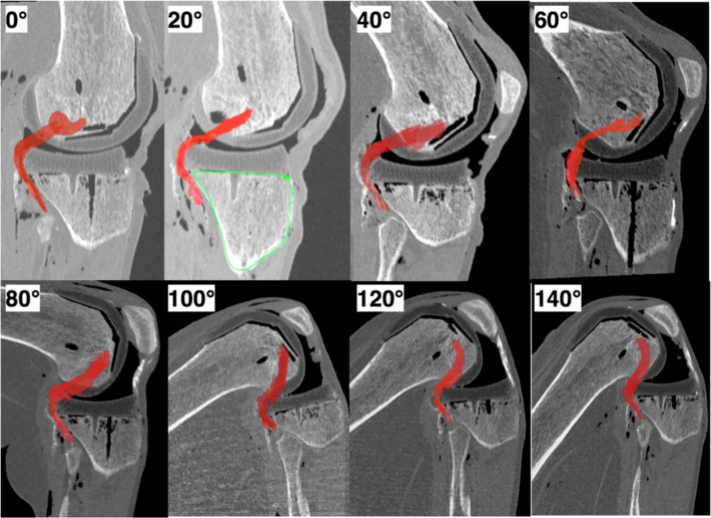
Sagittal view of the postoperative knee during the full range of flexion. The popliteus tendon is colored in red

Exploration from full extension to deep flexion appeared to be difficult with MRI. First, the small diameter of the MRI tube imposed a mid-shaft transection of the tibia and femur, which changed the quadriceps tension, creating a patella infera and induced a retraction of the hamstring muscles and the gastrocnemius. Second, beyond 90° a ‘mirror image’ appeared, which prevented full visualization of the knee. Third, MRI acquisition was time-consuming, requiring 30 min per sequence. The CT scans provided good quality images throughout the ROM (Fig. 7).

### Three-dimensional reconstructions

**Fig. 8:**
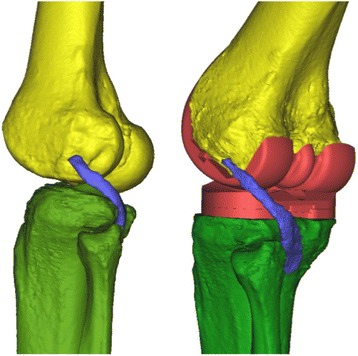
3D reconstructions of the knee before (*left*) and after (*right*) implantation of the TKA. The modification of the position of the popliteus tendon is clearly visible

3D reconstructions were done from group III, using CT scans on fresh-frozen specimens. Stereolithography (STL) files of the appropriate implant sizes were superimposed on the tibia and femur by manual matching, using the same landmarks. To compare the pre- and post-operative position of the soft tissues, the pre-operative Mimics® file was imported and matched with the post-operative file (Fig. 8).

## Discussion

The role of soft tissue impingement in residual pain after joint prostheses has been emphasized only recently. In Total Hip Arthroplasty (THA) the first ilipsoas impingements were described in 1995 by Trousdale (Trousdale et al. 1995), and in TKA, painful impingements between the Popliteus Tendon and the lateral condyle were described by Barnes and Scott in 1995 and by Allardyce et al. in 1997 (Allardyce et al. 1997; Barnes & Scott 1995). Generally speaking, the influence of component overhang on residual pain after TKA has been investigated only in the last decade (Bonnin et al. 2015; Bonnin et al. 2013; Mahoney & Kinsey 2010) and we can guess that the real rate of such impingements is underestimated due to the difficulties of clinical imaging. Better knowledge of interactions between soft tissues and implants would be critical to identify (Kazakin et al. 2014; Trousdale et al. 1995) and prevent impingements by improving surgical techniques (Kazakin et al. 2014; Trousdale et al. 1995) or implant design (Argenson et al. 2005; Vandenbussche et al. 2007).

To the authors’ knowledge, the precise relations between TKA implants and surrounding soft tissues have never been investigated during the full ROM. Several computational models using finite element analysis have been developed, that contribute to this understanding (Baldwin et al. 2012; Marra et al. 2015). These models provide a platform for researchers to simulate knee flexion in weight-bearing conditions, with a given implant. They allow experimental testing using subtle changes from one test to another, i.e. slight variations of implant positioning, sizing or design. However these models have several limitations: (i) they are mostly designed to quantify muscle, ligament, and knee joint contact forces and areas; (ii) they rely only on data obtained from one or few specimens (Baldwin et al. 2012; Marra et al. 2015); (iii) a limited number of soft tissues is modeled, i.e. patellar tendon, rectus femoris and vastus intermedius (Hoops et al. 2012) and (iv) their tracking during flexion-extension is only deduced from standard MRI in extension. Therefore, we intended to elaborate a protocol for soft tissue imaging around TKA, after implantation of commercially available prostheses, in realistic surgical conditions and on different human specimens. The goal was to provide images of the tracking of the soft tissues around the knee, before and after TKA, during the full range of flexion.

This work demonstrates that the use of implants made of acrylonitrile butadiene styrene and barium-free cement allows imaging of the implants without major scattering. While the study was originally intended to design and validate a method to image soft tissues around real metal and polyethylene implants, the authors were unable to overcome challenges caused by of image distortion and artifacts, despite repetitive involvement of engineers from the research and development department at the manufacturers of the scanners. The use of 3D printers provides the full range of sizes of many commercially-available prostheses and enables implantation in realistic conditions. The described CT technique should be used in the future to compare implants and to optimize their designs, with respect to interactions between implants and soft tissues. With the MRI protocol, neither the injection of embalming fluids nor the 20 °C temperature of the room modified significantly the conductivity of the cadaveric tissues and their magnetic resonance properties. The two main reasons for which we do not recommend MRI are poor image quality of the implants and difficulties in differentiation between soft tissues and implants (Fig. 2). Also the presence of ‘mirror images’ in deep flexion, probably due to the small weight of the specimens, prevented to investigate deep flexion. With the CT scan technique, a good visualization of the injected soft tissues and their relationships with the implants was obtained during the full range of knee flexion (Table 2).

When choosing the size and/or design of components in TKA, one of the goals is to match the cortical contour of the bone cut area, avoiding overhangs (Bonnin et al. 2015; Bonnin et al. 2013; Hitt et al. 2003; Mahoney & Kinsey 2010) but also optimizing the bone coverage (Berend et al. 2008), which may require some compromise (Martin et al. 2014). Consequently, morphometric analysis, upon which implants are designed, are frequently based on measurements at the bone-cut levels done in vitro (Kwak et al. 2007), in vivo (Hitt et al. 2003; Westrich et al. 1995) or based on CT scans (Bonnin et al. 2016; Bonnin et al. 2011). However it has been recently demonstrated that patients with slightly undersized components had better outcomes than patients with normosized implants and that matching the bone-cut contours is an over-simplification (Bonnin et al. 2013). We may assume that with several TKA implants, despite a good bone-implant fit at the resection planes, oversizing or ‘over-stuffing’ may occur in terms of volume.

The strength of the described CT scan protocol is that it provides precise 3D imaging of the soft tissues around TKA and may help to compare different implant designs, implant kinematics and also different joints. Its main limitation is that it required direct soft tissue injections via a lateral incision and an anteromedial arthrotomy, which can modify the normal anatomy. Another limitation is that it was not possible to quantify the clarity of soft tissues nor utility of each imaging technique, mainly because there was no existing ‘gold standard’ , but also because routine clinical assessments of soft tissues are chiefly qualitative or semi-quantitative. Finally, the images were acquired for this study without weight-bearing or natural muscle tensions, which can be simulated in finite element models. Nevetheless, by virtue of conformity of articular surfaces in TKA, we can assume that soft tissue contacts with bone and implant surfaces are similar in a weight-bearing situation.

## Conclusion

The experimental approach described in this study was successful in visualizing the real relationships between the soft tissues and the implants before and after TKA and throughout the range of motion. The coordinate system allows to localize precisely the different anatomic structures and to quantify changes due to prosthetic implantation. This protocol permits accurate analysis of soft tissue displacements following implantation of TKAs of different designs, sizes and alignments. A perspective could be to match the anatomic data obtained from this work with computational models.
