# Erratum to: Imaging the implant-soft tissue interactions in total knee arthroplasty

**DOI:** 10.1186/s40634-016-0065-1

**Published:** 2016-10-11

**Authors:** Michel P. Bonnin, Tom Van Hoof, Arnoud De Kok, Matthias Verstraete, Catherine Van der Straeten, Mo Saffarini, Jan Victor

**Affiliations:** 1 Centre Orthopédique Santy, 24 Av Paul Santy, Lyon, France; 2 Hopital Privé Jean Mermoz, 55 Av Jean Mermoz, 69008 Lyon, France; 3 UZ Gent, De Pintelaan, 185 Gent, Belgium; 4 Accelerate Innovation Management, Rue de Hollande 4-6, 1204 Geneva, Switzerland

## Erratum

Unfortunately, after publication of this article (Bonnin et al. 2016), it was noticed that the name of Mo Saffarini was incorrectly displayed as Moreno Saffarini. The corrected author list can be seen above and the original article has been updated to correct this error.
